# Alopecia as an Emerging Adverse Effect Associated With Glucagon-Like Peptide-1 (GLP-1) Receptor Agonists for Weight Loss: A Scoping Review

**DOI:** 10.7759/cureus.90021

**Published:** 2025-08-13

**Authors:** Ricardo Flaminio Rojas Lopez, Daniela Lynett Barrera, Maria Camila Amaya Muñoz, Maria Paula Saavedra Diaz

**Affiliations:** 1 Dermatology and Trichology, Dermahair Center, Floridablanca, COL; 2 Epidemiology, Dermahair Center, Floridablanca, COL; 3 General Practice, Dermahair Center, Floridablanca, COL

**Keywords:** alopecia, dermatology, glucagon-like peptide-1 receptor agonists, hair loss, weight loss

## Abstract

Alopecia has recently been reported as a potential emerging adverse effect associated with glucagon-like peptide-1 receptor agonists (GLP-1RAs), which are widely prescribed for weight loss. While gastrointestinal symptoms remain the most frequently documented adverse effects, an increasing number of reports describe hair loss events in patients treated with semaglutide, liraglutide, tirzepatide, and dulaglutide. This scoping review aimed to synthesize the available evidence regarding this potential association. A systematic literature search was conducted across PubMed, Scopus, Google Scholar, Cochrane, and the Latin American and Caribbean Literature on Health Sciences (LILACS) database through May 24, 2025. Nine studies met the inclusion criteria, comprising randomized clinical trials, cohort studies, and pharmacovigilance analyses. Most studies lacked dermatological diagnostic confirmation, and only one described the clinical pattern of alopecia, identifying telogen effluvium and androgenetic alopecia as the most frequent subtypes. The findings suggest that GLP-1RAs may alter the hair follicle cycle. More than 1,000 spontaneous cases have been reported in the U.S. Food and Drug Administration Adverse Event Reporting System (FAERS). Although a causal relationship cannot be confirmed, the recurrence of cases across diverse settings signals a potential safety concern that warrants clinical attention. Awareness of this possible effect may improve therapeutic adherence and prevent unnecessary diagnostic interventions. Further dermatological research is needed to better characterize the frequency, temporality, and underlying mechanisms of GLP-1RA-associated alopecia.

## Introduction and background

Alopecia is a common reason for consultation in dermatology and trichology, with a wide range of recognized etiologies, including drug-induced causes [1]. In recent years, a relatively unexplored safety signal has emerged: the development of alopecia in patients treated with glucagon-like peptide-1 receptor agonists (GLP-1RAs), approved for weight reduction [2,3].

These agents, such as semaglutide, liraglutide, dulaglutide, and tirzepatide, were initially developed for the treatment of type 2 diabetes mellitus, but have demonstrated significant clinical benefits in achieving sustained weight loss, leading to their approval for obesity management even in individuals without diabetes [4].

The most commonly reported adverse effects in clinical trials and post-marketing surveillance include gastrointestinal symptoms such as nausea, vomiting, diarrhea, and constipation [5]. However, alopecia has been noted in cohort studies with limited sample sizes [2,3] and pharmacovigilance analyses [6], with no comprehensive clinical characterization currently available to determine its prevalence, clinical features, or underlying pathophysiological mechanisms. Although infrequent, this adverse effect raises concern due to its potential impact on treatment adherence and patient quality of life.

Given the increasing use of GLP-1RAs in obesity treatment and the possible implications of alopecia on adherence, it is essential to systematically explore this association. Therefore, the aim of this scoping review is to synthesize and map the existing evidence regarding alopecia as a potential adverse event linked to GLP-1RA use for weight loss.

## Review

Methodology

This study was conducted as a scoping review following the Preferred Reporting Items for Systematic Reviews and Meta-Analyses extension for Scoping Reviews (PRISMA-ScR) guidelines. The guiding research question was: What is the available evidence on alopecia as an emerging adverse effect associated with the use of GLP-1 receptor agonists prescribed for weight loss?

Population, Intervention, Control, and Outcomes (PICO) Question

P (Population): patients receiving pharmacological treatment for weight loss; I (Intervention): use of GLP-1RAs; C (Comparator): not applicable; O (Outcome): alopecia.

Outcome Definition

The primary outcome was alopecia, defined as the "absence of hair from areas where it is normally present," according to the Medical Subject Headings (MeSH) term from the National Library of Medicine (NIH).

A scoping review was chosen instead of a systematic review because the topic represents an emerging area of clinical concern with limited and heterogeneous evidence. The available studies vary widely in design, quality, populations, and reporting of alopecia outcomes, which precludes conducting a quantitative synthesis or meta-analysis. Thus, the aim was to comprehensively map the existing literature, identify knowledge gaps, and generate hypotheses to guide future focused research.

Search Strategy

A systematic search was performed in the PubMed/MEDLINE, Scopus, Google Scholar, Cochrane, and Latin American and Caribbean Literature on Health Sciences (LILACS) databases, including articles published up to May 24, 2025. No relevant studies were found in Cochrane or LILACS. Articles in English or Spanish were included regardless of publication date. The search strategy combined MeSH and free terms related to “GLP-1 receptor agonist,” “liraglutide,” “semaglutide,” “tirzepatide,” “dulaglutide,” “exenatide,” “lixisenatide,” “alopecia,” “hair loss,” “overweight,” and “obesity” (Appendix).

Inclusion and Exclusion Criteria

Studies reporting hair loss in patients receiving GLP-1RAs for weight management were included, with no restrictions on age. Eligible study designs encompassed randomized controlled trials, observational cohort studies, pharmacovigilance analyses, prospective pilot studies, and grey literature documents.

For clinical studies, only those explicitly indicating weight loss, either with or without concomitant diabetes mellitus, as the primary treatment objective were included. Studies in which the stated indication was unrelated to weight management were excluded.

For pharmacovigilance studies, reports were considered regardless of treatment indication when such information was not specified, reflecting the inherent nature of spontaneous reporting systems. However, if the indication was clearly stated and unrelated to weight loss, the report was excluded.

Reviews, commentaries, editorials, animal studies, and studies not available in full text were also excluded.

Study Selection and Data Extraction

Two reviewers (MCAM, MPSD) independently screened the studies, first by evaluating titles and abstracts, followed by full-text review. Discrepancies were resolved by consensus with the participation of two senior reviewers (RFRL, DLB). A structured data extraction matrix was created using Google Sheets.

Assessment of Methodological Quality

Two reviewers (MCAM, MPSD) independently assessed the risk of bias of the included studies using version 2 of the Cochrane Risk of Bias tool for randomized trials (RoB 2) [7] and the ROBINS-E tool [8] for observational studies. Any discrepancies in bias assessment were resolved by consensus with the involvement of a senior reviewer (RFRL or DLB).

Results

**Figure 1:**
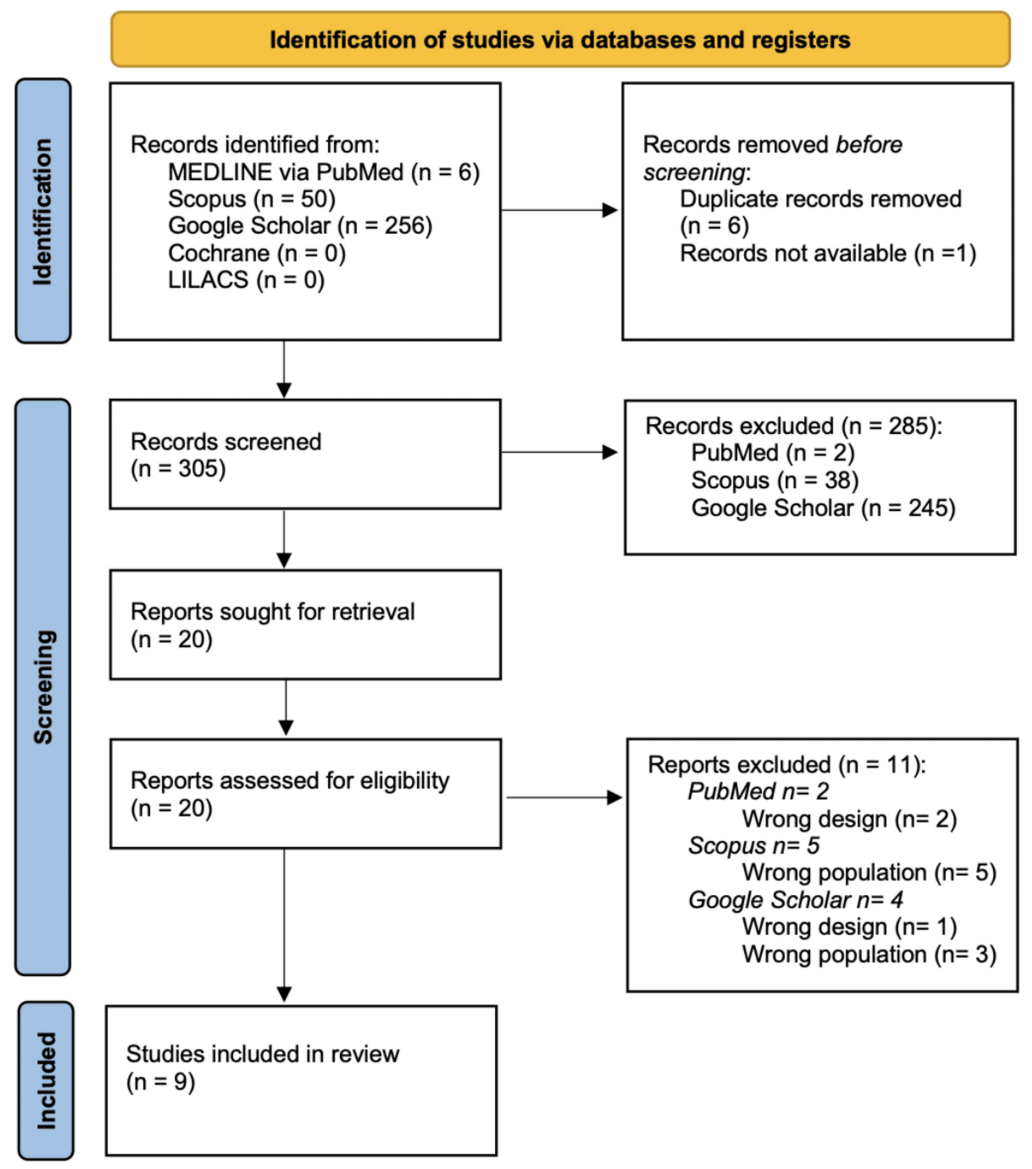
Preferred Reporting Items for Systematic Reviews and Meta-Analyses (PRISMA) study flow diagram

Nine studies were included in this scoping review following the systematic search across PubMed, Scopus, Google Scholar, Cochrane, and LILACS. Although five databases were searched, no eligible studies were identified in Cochrane or LILACS, which were therefore excluded from the final analysis. The selection and exclusion process is detailed in the PRISMA flow diagram (Figure 1).

The selected studies encompass a range of designs: four randomized, placebo-controlled clinical trials, one prospective pilot study, three retrospective pharmacovigilance studies, and one cohort study. This methodological heterogeneity reflects the emerging nature of the association between GLP-1RAs and alopecia and highlights a scientific field still under development.

The total number of patients treated with GLP-1RAs ranged from 19 to 1,896 per cohort, excluding aggregated pharmacovigilance records. The most frequently studied agents were semaglutide, liraglutide, dulaglutide, and tirzepatide. GLP-1RAs were prescribed for obesity management, with or without concomitant type 2 diabetes mellitus.

The reported frequency of alopecia varied widely across studies: from three cases in a pilot cohort of 19 patients, to 35 cases in a sample of 283 patients, and over one thousand reports in the FDA Adverse Event Reporting System (FAERS) retrospective adverse event analysis. Sex distribution was reported in six studies, revealing a predominance of female patients (63% to 78.6%). The mean age of affected individuals ranged from 40 to 56 years. Only one study included a pediatric population, with a mean age of 15.1 years [12].

Only one study provided clinical characterization of alopecia, identifying non-scarring forms - such as androgenetic alopecia (AGA) and telogen effluvium (TE) - as the most frequently observed patterns. Less commonly reported alopecias included frontal fibrosing alopecia, alopecia areata, and discoid lupus alopecia. In the same study, 91.4% of patients with alopecia had a personal history of hair loss [3].

The most frequently reported comorbidities among patients with alopecia were hypertension, dyslipidemia, obstructive sleep apnea, prediabetes, and cardiovascular disease. Table 1 summarizes the key characteristics of the included studies.

**Table 1:** Summary of the Characteristics of Included Studies GLP-1RA = glucagon-like peptide-1 receptor agonists; mg = milligrams; SC = subcutaneous; PO = oral administration; DLE = discoid lupus erythematosus; T2DM = type 2 diabetes mellitus; FAERS = FDA Adverse Event Reporting System; DAEN = Database of Adverse Event Notifications; COPD = Chronic Obstructive Pulmonary Disease; – = not reported.

Title	Author and year	Type of study	GLP-1RA used	Dose	Treatment duration	Indication for use	Total patients who received GLP-1RA	Number of patients treated with GLP-1RA who developed alopecia	Type of alopecia	Personal history of alopecia	Sex of patients treated with GLP-1RA	Age in years (mean)	Comorbidities
Glucagon-like peptide-1 receptor agonist medications and hair loss.	Burke et al., 2025 [3]	Retrospective cohort	Dulaglutide, Liraglutide, Semaglutide, Tirzepatide	-	-	Weight loss	283	35	19 (Androgenetic alopecia) 10 (Telogen effluvium) 3 (Unspecified hair loss) 4 (Unspecified alopecia) 6 (Other types of alopecia, including central centrifugal cicatricial alopecia, trichorrhexis nodosa, frontal fibrosing alopecia, alopecia areata, discoid lupus erythematosus, folliculitis)	32 cases	185 women (65.4%), 98 men (34.6%)	56.3	-
A Retrospective Comparative Analysis of Cutaneous Adverse Reactions in GLP-1 Agonist Therapies.	Daniel et al., 2025 [9]	Retrospective observational study based on pharmacovigilance data (FAERS)	Semaglutide, Dulaglutide, Tirzepatide, Liraglutide, Exenatide, Lixisenatide	-	-	Weight loss and type 2 diabetes management	-	1,162 (239 semaglutide, 239 dulaglutide, 155 tirzepatide, 251 liraglutide, 278 exenatide, 0 lixisenatide)	-	-	-	-	-
Pilot study to test the safety, tolerability and feasibility of dulaglutide during a low-energy diet for weight loss and improved glycaemic control.	Shand et al., 2023 [2]	Pilot study: prospective, single-arm, open-label	Dulaglutide	1.5 mg s.c once weekly	14 weeks	Weight loss and type 2 diabetes management	19	3	-	1 case	9 women (47%), 10 men (53%)	40.2	Arterial hypertension
Tirzepatide Once Weekly for the Treatment of Obesity.	Jastreboff et al., 2022 [10]	Randomized, double-blind, placebo-controlled clinical trial	Tirzepatide	5 mg, 10 mg, and 15 mg s.c once weekly	72 weeks	Weight loss	630 doses of 5 mg, 636 doses of 10 mg and 630 doses of 15 mg	99 cases: 32 (5 mg), 31 (10 mg), 36 (15 mg)	-	-	5 mg: 426 women (67.6%), 204 men (32.4%) 10 mg: 427 women (67.1%), 209 men (32.9%) 15 mg: 425 women (67.5%), 205 men (32.5%)	45.6 (5 mg), 44.7 (10 mg), 44.9 (15 mg)	Hypertension, prediabetes, dyslipidemia, sleep apnea, cardiovascular disease
Tirzepatide after intensive lifestyle intervention in adults with overweight or obesity: the SURMOUNT-3 phase 3 trial.	Wadden et al., 2023 [11]	Randomized, double-blind, placebo-controlled clinical trial	Tirzepatide	Maximum tolerated dose: 10 or 15 mg s.c once weekly	72 weeks	Weight loss	287	20	-	-	81 women (63.1%), 106 men (36.9%)	45.4	Hypertension, dyslipidemia, obstructive sleep apnea, cardiovascular disease, polycystic ovary syndrome, osteoarthritis, anxiety/depression, asthma or COPD, gout, non-alcoholic fatty liver disease
Liraglutide in an Adolescent Population with Obesity: A Randomized, Double-Blind, Placebo-Controlled 5-Week Trial to Assess Safety, Tolerability, and Pharmacokinetics of Liraglutide in Adolescents Aged 12-17 Years.	Danne et al., 2017 [12]	Randomized, double-blind, placebo-controlled clinical trial	Liraglutide	Initial dose of 0.6 mg, titrated weekly by 0.6 mg increments to a maximum dose of 3.0 mg daily	5 weeks	Weight loss	14	1	-	-	11 women (78.6%) and 3 men (21.4%)	15.1	-
Alopecia associated with the use of semaglutide and tirzepatide: A disproportionality analysis using the FDA adverse event reporting system (FAERS) from 2022 to 2023.	Godfrey et al., 2024 [6]	Retrospective observational study based on pharmacovigilance data (FAERS)	Semaglutide, tirzepatide, liraglutide, dulaglutide, exenatide	-	-	-	-	469 (199 semaglutide, 179 tirzepatide, 20 liraglutide, 65 dulaglutide, 6 exenatide)	-	-	-	-	-
Risk of Suicide, Hair Loss, and Aspiration with GLP1‐Receptor Agonists and Other Diabetic Agents: A Real‐World Pharmacovigilance Study.	Nakhla et al., 2024 [13]	Retrospective observational study based on pharmacovigilance data (FAERS, EudraVigilance, VigiBase, DAEN)	Semaglutide, liraglutide, dulaglutide, exenatide, lixisenatide	-	-	-	-	4.888 (FAERS: 1.407, EudraVigilance: 3.360, VigiBase: 117, DAEN: 4)	-	-	-	-	-
Oral semaglutide 50 mg taken once per day in adults with overweight or obesity (OASIS 1): a randomised, double-blind, placebo-controlled, phase 3 trial.	Knop et al., 2023 [14]	Randomized, double-blind, placebo-controlled clinical trial	Semaglutide	50 mg orally per day	68 weeks	Weight loss	334	23	-	-	247 women (74%) and 87 men (26%)	49	Hypertension, dyslipidemia, obstructive sleep apnea, cardiovascular disease

Although clinical characterization was limited in most studies, alopecia was consistently reported as an adverse event across various clinical contexts and GLP-1RA agents. While the available evidence does not yet support a definitive causal relationship, it raises a relevant safety signal that merits specialized follow-up.

Risk of Bias Assessment

**Figure 2:**
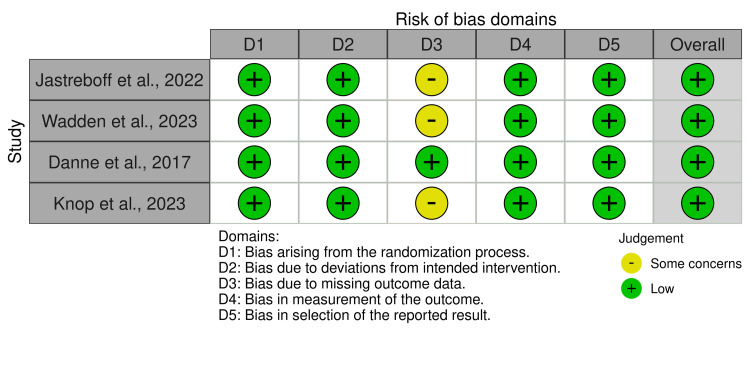
Risk of Bias Assessment for Randomized Clinical Trials Using the RoB 2 Tool [10-12,14] RoB 2 tool [7].

**Figure 3:**
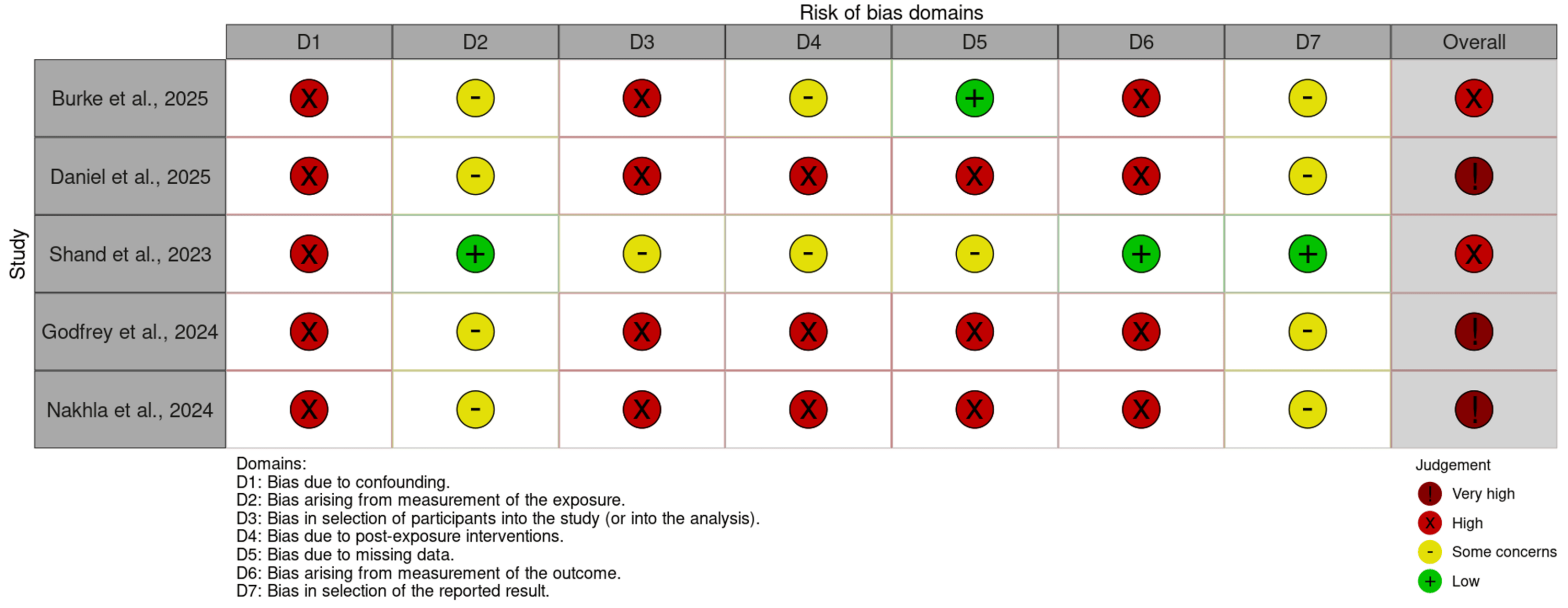
Risk of Bias Assessment for Observational Studies Using the ROBINS-E Tool [2,3,6,9,13] ROBINS-E tool [8].

The risk of bias assessment for the included studies is presented in Figure 2 and Figure 3. The randomized clinical trials were rated as having a low risk of bias, as they demonstrated clear randomization, appropriate blinding, and objective outcomes measured in a standardized manner.

In contrast, all observational studies were rated as having a high or very high risk of bias, due to lack of confounding control, selection bias (as only patients with reported adverse events were included), subjectively assessed outcomes without clinical validation, and reliance on pharmacovigilance databases containing incomplete information and susceptible to selective reporting.

Discussion

This is the first scoping review to address the potential association between GLP-1RAs and alopecia as an emerging adverse effect in the context of weight loss. The interest in this review stemmed from clinical observations in dermatology and trichology, where cases of alopecia were identified following the initiation of these medications. Given the growing use of GLP-1RAs in the management of obesity, it is essential to explore dermatological side effects that, although infrequently reported, can significantly affect patients' quality of life and, consequently, treatment adherence [15].

Available evidence is limited to primary studies, randomized trials, retrospective cohorts, and pharmacovigilance analyses with no confirmed causal relationship or defined pathophysiological mechanisms. A recent study (July 2025) reported alopecia as an uncommon adverse event (one to five cases per 1,000 treated patients) [16], which may reflect underreporting due to lack of awareness of this association or low clinical suspicion.

This review identified the occurrence of alopecia across diverse clinical settings, suggesting a potential safety signal warranting further investigation. Notably, most studies did not include dermatological diagnostic tools such as trichoscopy or scalp biopsy, limiting accurate characterization of the alopecia subtype and its potential etiology. In many cases, however, a biopsy is not necessary for diagnosis, underscoring the importance of clinical suspicion and targeted patient history as key tools to avoid underreporting.

Only one of the included studies provided clinical characterization of alopecia subtypes, identifying primarily non-scarring forms such as TE and AGA [3]. TE has been classically associated with acute metabolic stress and with metabolic and nutritional changes resulting from rapid weight loss [17]. On the other hand, GLP-1RAs have been proposed to alter the hair follicle cycle [3,17], which, in predisposed individuals, could promote the development of TE or AGA. The same study by Burke et al. [3] also reported isolated cases of frontal fibrosing alopecia and discoid lupus erythematous which could suggest the possible involvement of immune-mediated mechanisms in susceptible patients.

Another relevant observation is that the temporal relationship between treatment initiation and the onset of alopecia was not reported systematically, which hinders the ability to draw solid conclusions about causality. This lack of detail prevents an accurate assessment of the time-dose relationship needed to determine the potential role of the drug. Furthermore, uncertainty remains as to whether symptoms resolved after discontinuation of the treatment, a clinically relevant aspect. While some studies documented the administered dose, others did not, limiting the ability to identify clear relationships between dose and the occurrence or severity of the adverse event.

From a pharmacovigilance perspective, the FAERS analysis highlights the scope of the phenomenon, with over 1,000 spontaneous reports of alopecia associated with GLP-1RA use. However, this type of data source is subject to inherent biases such as selective reporting and does not allow for the calculation of adjusted incidence rates.

Some authors have begun exploring emerging dermatological manifestations related to these drugs from different perspectives. Paschou et al. and Ridha et al. proposed a possible link between GLP-1RAs and accelerated skin aging via oxidative stress and altered dermal metabolism, mechanisms that could potentially extend to the hair follicle [18,19]. Desai et al. also emphasized the need to investigate dermatologic events beyond those classically recognized [20]. From a clinical pharmacology standpoint, Gorgojo-Martínez et al. provided recommendations for monitoring gastrointestinal side effects of GLP-1RAs but also highlighted that proper management of adverse outcomes can significantly improve treatment adherence and persistence, ultimately enhancing quality of life [5]. This observation is particularly relevant in the case of alopecia, a potentially underestimated but highly impactful adverse effect. Meanwhile, the review by Tchang et al. [4] on GLP-1RA safety profiles did not include skin-related events, pointing to a historical gap in the surveillance of such adverse effects.

Despite the inherent limitations of scoping reviews (such as the inability to perform quantitative synthesis and the heterogeneity of study designs) and the low methodological quality of included observational studies, this review offers a systematic and rigorous overview of the available literature. A structured assessment of methodological quality was applied, and gray literature was included to enhance coverage. However, the lack of data on event timing, alopecia subtype, patient history, and outcomes following drug discontinuation limits the analysis and leaves key questions unanswered for future investigation.

In this context, we propose an integrative pathophysiological hypothesis to explain alopecia in patients using GLP-1RAs. Rapid weight loss induced by these agents generates acute metabolic stress, which may trigger telogen effluvium, a transient, non-scarring form of hair loss well documented in the context of bariatric surgery, systemic illness, or strict hypocaloric diets [3,21]. Additionally, some studies suggest that these agents could also act as triggers for androgenetic alopecia in predisposed individuals, partly through hormonal modulation during weight loss [3].

More speculatively, indirect mechanisms have been proposed involving the expression of GLP-1 receptors in dermal fibroblasts and adipose-derived stem cells. Their activation may alter local cytokine production, increase oxidative stress, or disrupt cellular bioenergetics. However, these models are better described in the context of accelerated skin aging linked to GLP-1RA use, a phenomenon popularly known as “Ozempic face” [8,20], and have yet to be specifically validated in relation to alopecia [17].

This preliminary hypothesis may serve as a foundation for future clinical studies with a dermatologic focus, including systematic trichologic characterization and longitudinal follow-up.

This review highlights the urgent need for systematic clinical research that includes alopecia as a safety endpoint in prospective trials. Standardized dermatologic evaluations should be incorporated, including targeted medical history, trichoscopy, clinical classification of alopecia subtypes, and scalp biopsy when indicated, along with structured longitudinal monitoring. It is also crucial to improve pharmacovigilance systems to enhance the detection and clinical characterization of dermatologic adverse events with greater precision.

## Conclusions

Alopecia has been repeatedly reported as a potential emerging and undercharacterized adverse effect in patients treated with GLP-1 receptor agonists prescribed for weight loss. While the available evidence is limited and heterogeneous, the recurrence of cases across different clinical contexts suggests a relevant safety signal that deserves attention. Although a conclusive causal relationship cannot be established, the association carries meaningful clinical implications, particularly concerning treatment adherence and quality of life.

This review offers a first comprehensive mapping of the existing literature and underscores the need for future investigations incorporating systematic dermatologic characterization, precise temporal assessment, and longitudinal follow-up. Furthermore, the proposed pathophysiological hypothesis opens a novel line of research into the metabolic, hormonal, and dermatologic impact of these agents, contributing to a more holistic understanding of their effects beyond weight control.

## Appendices

Appendix - search strategy (PubMed/MEDLINE)

A systematic search was conducted in PubMed/MEDLINE on May 24, 2025, using the following combination of MeSH terms and free-text terms with Boolean operators:

("Glucagon-Like Peptide 1 Receptor Agonists"[MeSH Terms] OR "glp 1 receptor agonist*"[Title/Abstract] OR "liraglutide"[Title/Abstract] OR "semaglutide"[Title/Abstract] OR "tirzepatide"[Title/Abstract] OR "dulaglutide"[Title/Abstract] OR "exenatide"[Title/Abstract] OR "lixisenatide"[Title/Abstract]) AND ("Alopecia"[MeSH Terms] OR "Alopecia"[Title/Abstract] OR "hair loss"[Title/Abstract]) AND ("Overweight"[MeSH Terms] OR "Overweight"[Title/Abstract] OR "Obesity"[MeSH Terms] OR "obes*"[Title/Abstract] OR "Weight Loss"[MeSH Terms] OR "Weight Loss"[Title/Abstract])

This strategy was adapted for the other databases included in the review.
